# REG3A and IL22 have opposite effects on fat accumulation in the liver in a high-fat diet

**DOI:** 10.1038/s41598-024-84700-w

**Published:** 2025-09-30

**Authors:** Esther Borras Nogues, Sandrine Auger, Alisson Agus, Aurélia Bruneau, Florian Chain, Léa Graci, Philippe Langella, Jean-Marc Chatel

**Affiliations:** INRAE, AgroParisTech, UMR1319 MICALIS, Université Paris Saclay, Jouy en Josas, 78352 France

**Keywords:** Applied microbiology, Metabolic disorders

## Abstract

IL22 is a cytokine with many immunoregulatory effects. It also promotes mucus production, cell proliferation and wound healing, making it a key component of intestinal health. An increasing body of studies highlight the potential of IL22 as a therapeutic drug against NAFLD through the induction of antimicrobial peptides, especially of the Reg3 lectins family, and the regulation of gut microbiota. However, adverse reactions to this molecule in long term administration are little-studied. In this study, we gave daily doses of recombinant *L. lactis* delivering DNA vaccine for REG3A and IL-22 production to mice fed with a butter-based high fat diet (milk-fat diet or MFD). We aim to compare the effects of REG3A and IL-22 expression by the epithelial cells on the development of obesity-related conditions and the composition of the microbiota. We show that they have opposite effects on fat accumulation in the liver and insulin resistance development despite inducing a similar shift in fecal microbiota composition. Here, we provide evidence that daily long term administration of IL22 can aggravate NAFLD by mechanisms independent from gut microbiota.

**Supplementary Information:**

The online version contains supplementary material available at 10.1038/s41598-024-84700-w.

## Introduction

The consumption of high quantity of sugar and fat in western diet leads to an increased prevalence of obesity. Such diets were shown to modulate the intestinal microbiota in patients and induce chronic low-grade inflammation, which participates in the pathogenesis of the metabolic syndrome. Epidemiological data show that obesity leads to an increased risk of developing Inflammatory Bowel Diseases (IBD). These results were replicated in mice; in laboratory conditions, high-fat diets exacerbate colitis in chemically induced and spontaneous models. The immune system response to fat in the gut and consequent modulation of the gut microbiota through different mechanisms of action have a causal link with the development of metabolic disorder as shown in an experiment which resulted in increased insulin sensitivity in patients receiving intestinal microbiota transfer from lean donors^1^. Interplay between gut microbiota, their metabolites and inflammatory markers modulate the development of Non-Alcoholic Fatty Liver disease (NAFLD). Furthermore, the administration of probiotics improve inflammatory markers in NAFLD patients^2^. In mice model, microbiota transplant from obese donor to lean recipient induces obesity^3^. Obesity results from a complex interaction between metabolic, inflammatory, microbial, and environmental factors, where REG3A and IL-22 play a partial role. Their role is crucial in understanding how obesity affects the intestinal barrier and triggers a cascade of metabolic and inflammatory dysfunctions.

REG3A (and its murine homolog Reg3γ) is an antimicrobial peptide (AMP) critical to gut homeostasis as it maintains the spatial segregation of bacteria and host cells in the gut^4^. REG3A’s role in obesity is still highly debated. In mice, the production of Reg3γ by Paneth cells is reduced after 12 weeks of high-fat diet^5^. It should be noted that microbial shift precedes AMPs downregulation and both precede circulating inflammatory cytokines levels alterations, which suggests that AMPs expression in obesity is not the initial cause of microbial shift but might play a role in maintaining dysbiosis and establishing chronic inflammation in obese patients^5^. Another study found contradictory results and showed that overexpression of Reg3γ spontaneously leads to increased weight under normal diet conditions^6^.

IL-22 is an immunomodulatory cytokine whose induction is impaired in obese patients. It is a member of the IL-10 related cytokine family and its receptors are highly expressed in epithelial cells of the gastrointestinal tract. IL-22 signaling induces the production of antimicrobial peptides, such as the Reg3 lectins family, promotes cell proliferation and wound repair^7–10^. It also promotes mucins production through STAT3 activation. Its role in obesity and inflammation is still debated. While it has anti-inflammatory and wound repair effects in the gut, studies have found CD4 + T cells from type 2 diabetes patients had increased production of IL-22 which in turn upregulated IL-1β production by adipose tissue macrophage and amplified inflammation^10^. The different pro- or anti-inflammatory properties of IL-22 depends on its regulation by other factors such as IL-17 A or IFN-γ^8^. Nevertheless, numerous studies have found exogenous administration of IL-22 in leptin-deficient and HFD feed mice reverses many symptoms of the metabolic syndrome, suppresses ER stress and inflammation^11–13^ although a 2015 study showed no effect of IL-22 administration or IL-22 genetic deletion on HFD-induced obesity and insulin resistance^14^.

The microbiota has a strong impact on the development of the immune system and IL-22 production is lacking in germ-free mice. IL-22 in turn influences the composition of microbiota through its modulation of mucins and antimicrobial peptides^15,16^. IL-22 deficiency in mice is associated with reduced abundance of some bacterial genera such as *Lactobacillus*,* Bacteroides* and *Ruminococcus*^15^.

Live bacteria have gained great interest as potential therapeutic vectors as they allow in situ production of molecules of interest. Multiple food grade bacteria have been developed as factories for the development of therapeutic proteins such as IL-2, IL-6 ^17^ or IL-10 and have been demonstrated to be biologically containable^18^. In 2020 J. Oh et al. found that administration of *Lactobacillus reuteri* expressing IL-22 reduced fatty liver disease in a high-fat diet mice experiment^19^. *L. lactis* MG1363 has also been genetically engineered with plasmid vectors using the cytomegalovirus promoter (pCMV) to deliver DNA vaccine to eukaryotic cells and induce production of the protein of interest in in vitro and in vivo assays^20–22^. Recently, the induction of IL-22 expression through intragastric administration of *L. lactis* engineered to deliver such a plasmid alleviated post infectious irritable bowel syndrome symptoms in mouse by restoring gut permeability, reduced colonic sensitivity and improved mice welfare^20^.

In this study, we compare long time administration of IL-22 and REG3A in the context of HFD. These mice typically develop a range of metabolic and obesity-related diseases such as weight gain, insulin resistance and hepatic steatosis. We showed that IL-22 and REG3A have opposite effects on fat accumulation in the liver and insulin resistance development despite inducing a similar shift in fecal microbiota composition.

## Results

### REG3A slightly increases the protective effect of *L. lactis* against weight gain

**Fig. 1:**
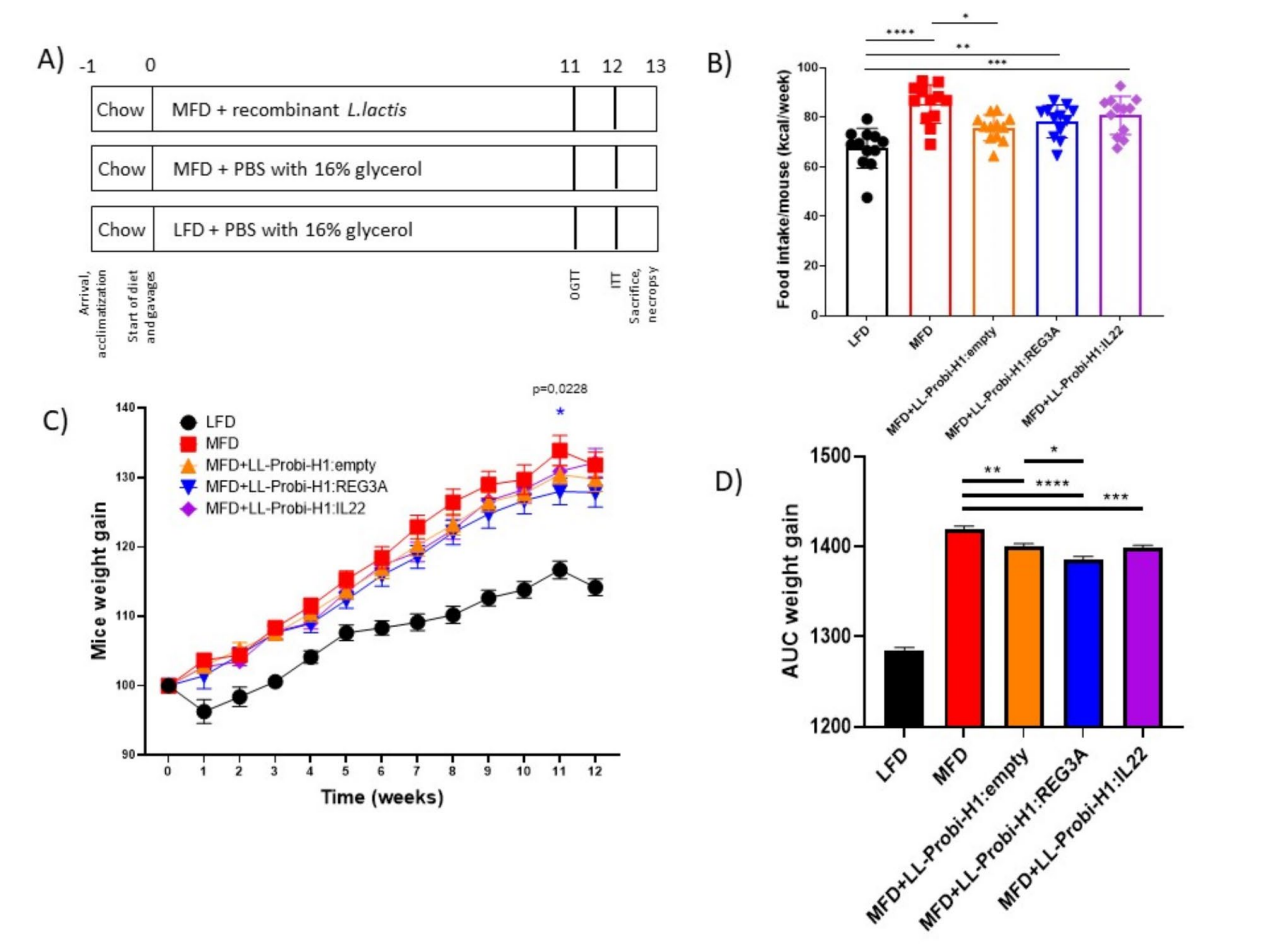
Effects of REG3A and IL22 on weight gain and food intake in mice. (**A**) Overview of the protocol. Mice were fed a milk-fat diet or low-fat diet and treated with or without recombinant *L. lactis* for over 12 weeks and sacrificed over the course of the 13th week. (**B**) Food intake (shown as mean in kcal/mouse per week) and (**C**) Weight development were controlled weekly. Body weight is represented as weight gain compared to first week. Significant differences were checked with repeated measure two-way ANOVA with multiple comparisons using Tukey’s post-hoc test. Significance is shown against MFD control group. (**D**) Area under curve of weight gain was calculated and significance was checked using one-way ANOVA with Tukey’s post-hoc test. Data represent mean ± SEM. *P < 0.05, **P < 0.01, ***P < 0.001, ****P < 0.0001. *n* = 16 mice per group.

Food intake was significantly increased in all groups fed with MFD except LL-Probi-H1:empty which eat significantly less (mean = 75,74 kcal/mice/week) than control MFD mice (mean = 85,39; pval = 0.0138, Fig. 1B). We note that all groups who received recombinant *L. lactis* tended to have a lower food intake compared to the control group despite the lack of statistical significance (LL-Probi-H1:REG3A = 78.39, pval = 0.1305; LL-Probi-H1:IL-22 = 80,77, pval = 0.5133). Body weight gain was significantly reduced in LL-Probi-H1:REG3A group compared to MFD control group 11 weeks after the start of the experiment (pval = 0.0228, Fig. 1C) but was not significantly different from the other LL-Probi-H1 groups. Area under curve of body weight gain shows that REG3A significantly reduces body weight gain compared to *L. lactis* administration (pval = 0.0186, Fig. 1D).

### REG3A and IL-22 treatments alter insulin tolerance in different ways

**Fig. 2:**
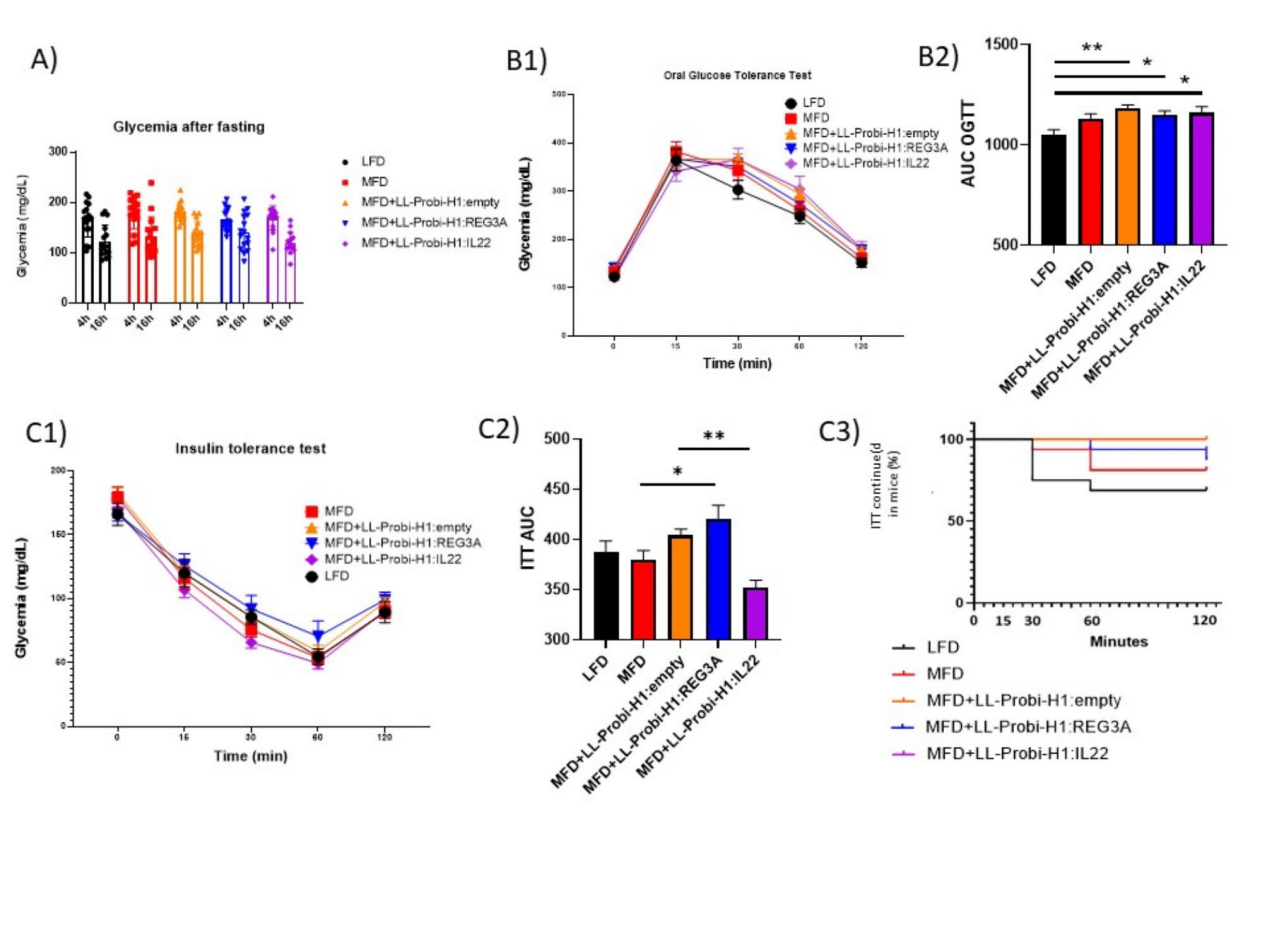
Effects of REG3A and IL22 on glucose regulation of MFD-fed mice. (**A**) Plasma glucose levels after 4 and 16 h fasting at week 11 and 12 respectively. (**B**) Blood glucose values during oral glucose tolerance test at week 11. (**C**) Insulin tolerance test at week 12. Area under curve of glucose concentrations were calculated for OGTT and ITT (**B2, C2**). ITT interruption due to low glycemia is represented as a survival curve (**C3**). Significance was checked using one-way ANOVA with Tukey’s post-hoc test. Data represent mean ± SEM. *P < 0.05, **P < 0.01, ***P < 0.001, ****P < 0.0001. *n* = 16 mice per group.

Oral glucose tolerance test and insulin tolerance test were respectively performed at week 11 and week 10 (Fig. 1A). Blood glucose levels after 4 and 16 h of fasting was not different between groups (Fig. 2A).

For OGTT, glycemia was slightly higher in MFD-fed groups compared to LFD control group but not significantly (Fig. 2B1) signaling they were still in prediabetic state. Area under curve of OGTT measurements was significantly higher for mice who received *L. lactis* treatments (Fig. 2B2). We found no significant difference in plasma insulin level between groups after 16 h of fasting or 30 min after glucose stimulation. Because LFD group insulin fasting levels were abnormally high and variable, we cannot exclude contamination of our samples occurred (supplementary Fig. 1).

For ITT, we found no significant difference in glycemia between our groups (Fig. 2C1). Area under curve of ITT measurements were higher in LL-Probi-H1:REG3A treated mice compared to MFD control group and lower in LL-Probi-H1:IL-22 treated mice compared to LL-Probi-H1:empty groups (Fig. 2C2). ITT had to be interrupted in 5 mice in the LFD group, 2 mice in the MFD control group, 2 mice in the LL-Probi-H1:REG3A group and 1 mouse in the IL-22 group. The LL-Probi-H1:empty group was the only one were all measurements could be done. Some mice showed signs of distress despite acceptable levels of blood glucose and ITT measurement had to be interrupted (Fig. 2C3).

### IL-22 aggravates fat accumulation and damage in the liver of MFD-fed mice

**Fig. 3:**
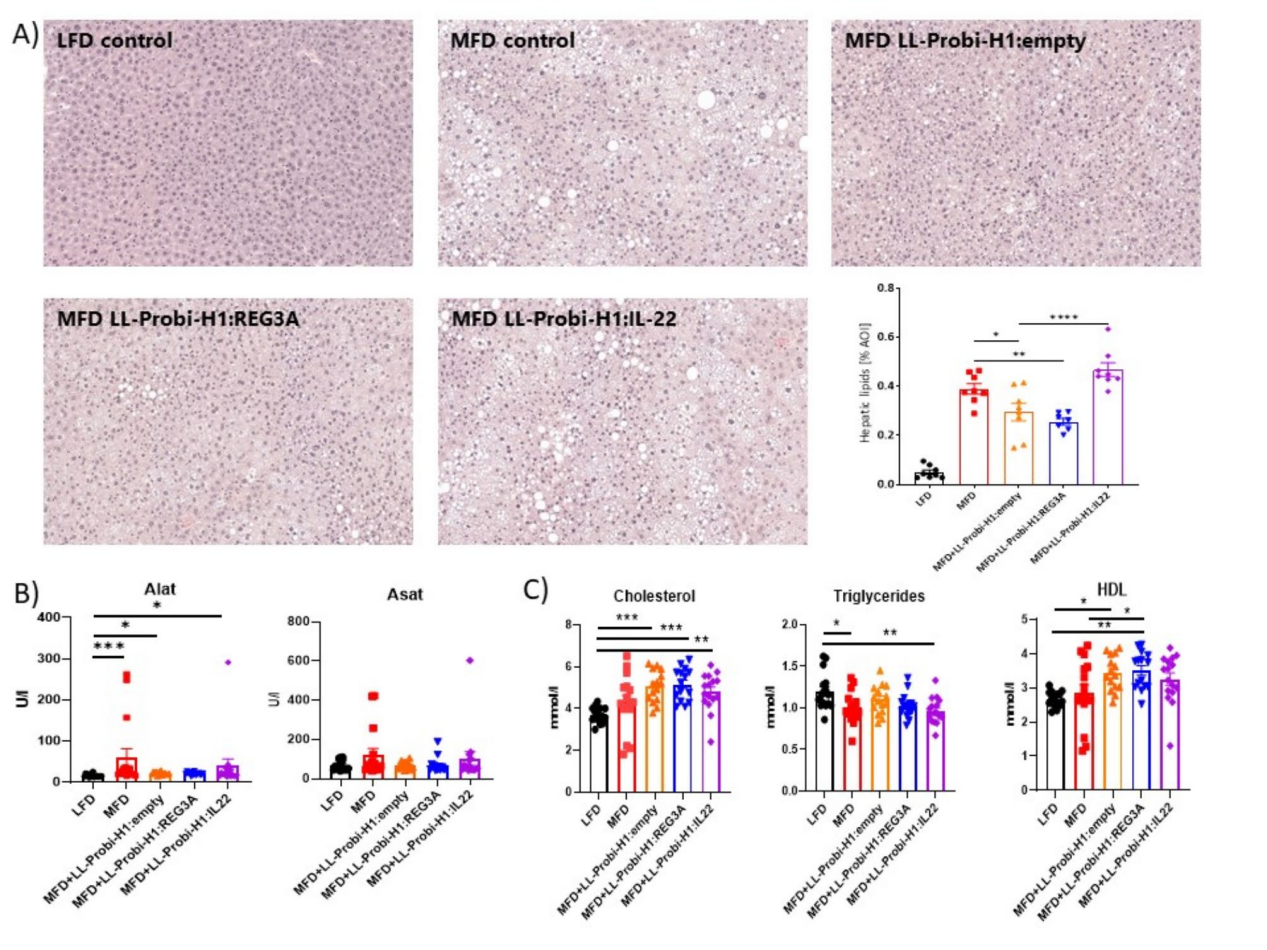
Effects of REG3A and IL22 on liver physiology. (**A**) Hepatic fat accumulation, (**B**) Circulating transaminases levels and (**C**) Circulating cholesterol and fatty acids levels 12 weeks after HFD and treatment started. Significance was checked using one-way ANOVA with Turkey’s post-hoc test when normality could be assumed or Kruskal-Wallis with Dunn’s post-hoc test otherwise. Data represent mean ± SEM. *P < 0.05, **P < 0.01, ***P < 0.001, ****P < 0.0001. *n* = 16 mice per group for blood measurements and *n* = 8 mice per group for histological examination.

Lipid accumulation (Fig. 3A) was greatly increased in all MFD fed-mice (mean area of interest = 0.39) compared to LFD control (mean AOI = 0.05). Administration of *L. lactis* in itself significantly reduces fat accumulation in the liver compared to MFD control (mean AOI = 0.29; pval = 0.0233). REG3A-treated mice have similarly lowered fat in the liver (mean AOI = 0.26; pval = 0.0016). IL-22 seems to completely reverse this effect and strongly increases fat accumulation in the liver compared to LL-Probi-H1:empty (mean AOI = 0.47; pval < 0.0001).

Plasma levels of alanine aminotransferase (ALAT) was increased significantly in all MFD-fed groups (pval : MFD = 0.0003; empty = 0.0126; IL-22 = 0.0222) compared to LFD but LL-Probi-H1:REG3A (pval = 0.07) (Fig. 3B). We note that 3 outliers are present in the MFD control group (with ALAT levels at 157, 248 and 262U/L respectively) and one outlier in the IL-22 group (ALAT level at 291U/L). We chose to keep them in the analysis as they reflect physiological damage of the liver after long-term MFD feeding. We also performed a one-way ANOVA analysis after removing these outliers (data not shown) but it did not impact which groups where significantly different from each other. Aspartate aminotransferase (ASAT) did not significantly differ between groups but we note that the trends are similar with ALAT levels and outliers appearing on the graph correspond to the same mice.

On the contrary, triglyceride (TG) levels are significantly decreased in MFD control and LL-Probi-H1:IL22 groups compared to LFD control (Fig. 3C). Cholesterol levels are significantly higher in all MFD-fed groups except for the control group due to 3 outliers which had abnormally low HDL and total cholesterol levels. We note that these outliers are the same mice which had extremely high ASAT (> 200 U/L) and ALAT (> 100 U/L) plasma levels (Fig. 3B) and suppose their cholesterol levels crashed down due to liver failure. One mice from the LL-Probi-H1:IL22 group had similarly low cholesterol levels and very high ASAT and ALAT levels. Removal of these mice from the statistical analysis changes the significance level against LFD control (LFD against MFD pval = 0.001 after removal of the 3 outliers). HDL levels trends coincide with total cholesterol levels although LL-Probi-H1:REG3A-treated mice have significantly higher HDL than MFD mice when all mice are included in the analysis (Fig. 3C). If outliers are removed, this difference disappears and all MFD-fed mice have significantly higher HDL levels than LFD-fed mice.

### MFD reduces fecal microbiota richness

**Fig. 4:**
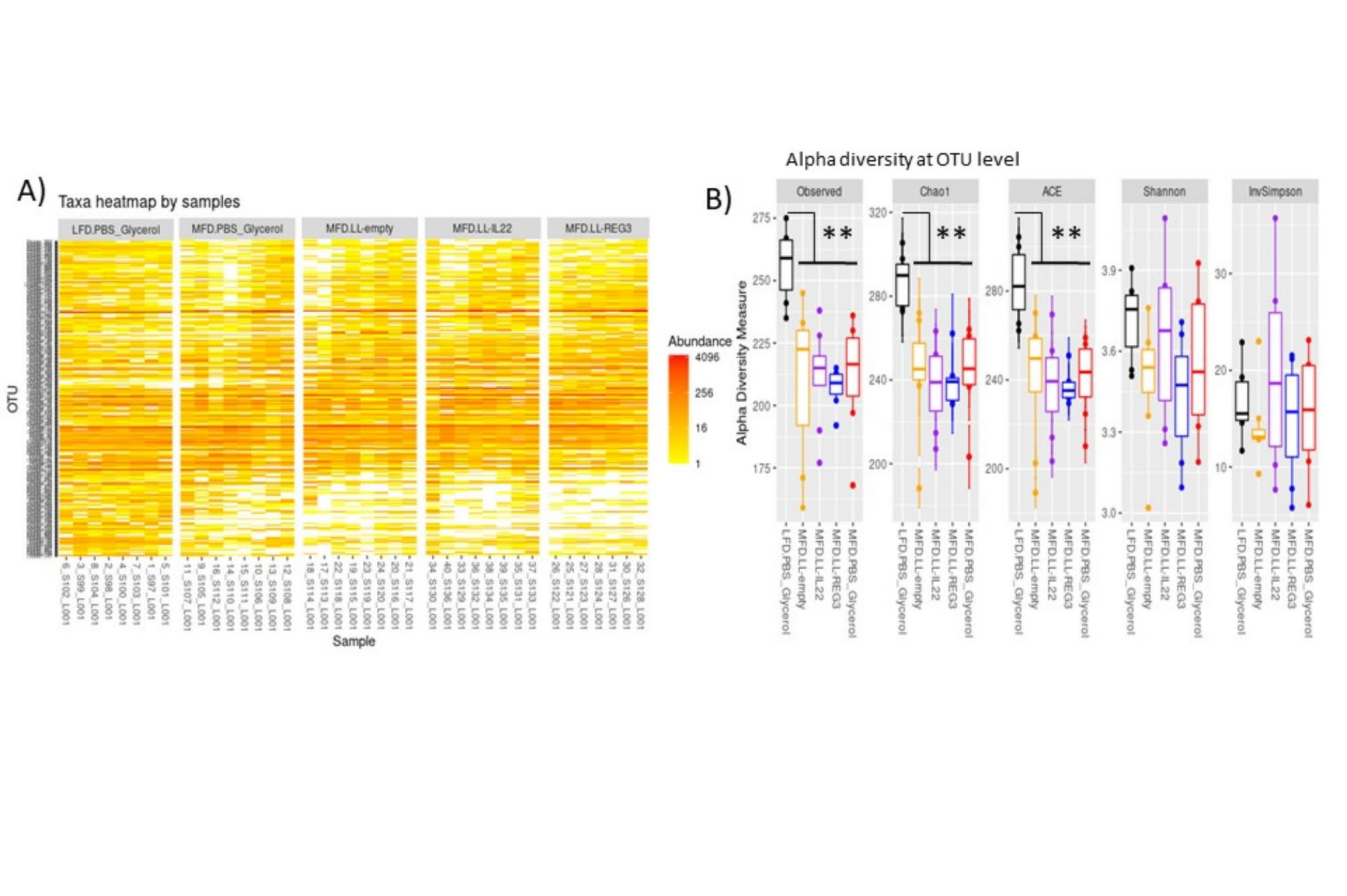
Milk-fat diet and recombinant *L.lactis* gavage effect on fecal microbiota composition. (**A**) Heatmap of the 250 most abundant OTUs in samples. (**B**) Alpha diversity at OTU level of samples by group represented in mean ± SD. *P < 0.05, **P < 0.01, ***P < 0.001, ****P < 0.0001. *n* = 8 mice per group. One mouse was removed from the analysis because of low OTU count compared to others (< 10000).

We investigated the composition of fecal microbiota after 12 weeks of MFD and treatment. We obtained a total of 2,121,530 sequences with an average of 53,038 ± 19,205 reads per sample. One sample was removed from the final analysis due to insufficient sequences count (9861 total reads and < 100 OTU sequenced). A total of 341 OTUs were detected. Five phyla (32 families and 70 genera) were represented in our samples: Bacteroidota, Firmicutes, Desulfobacterota, Proteobacteria and Actinobacteria. There was a great loss of OTU richness in MFD-fed mice compared to LFD, especially in the low abundance OTUs as can be seen on the heatmap (Fig. 4A) and alpha-diversity tests (observed, Chao1 and ACE indexes pval < 0.01, Fig. 4B). We note that Probi-H1:REG3A-treated mice had less variability in richness between samples than other groups. Intra-variability was not significantly impacted (Shannon and Inverse Simpson indexes). No significant difference in alpha-diversity was observed between MFD-fed treated and untreated mice (Fig. 4B).

### Gut microbiota composition correlates with diet, *L. lactis* treatment and liver damage at OTU and genus levels

**Fig. 5:**
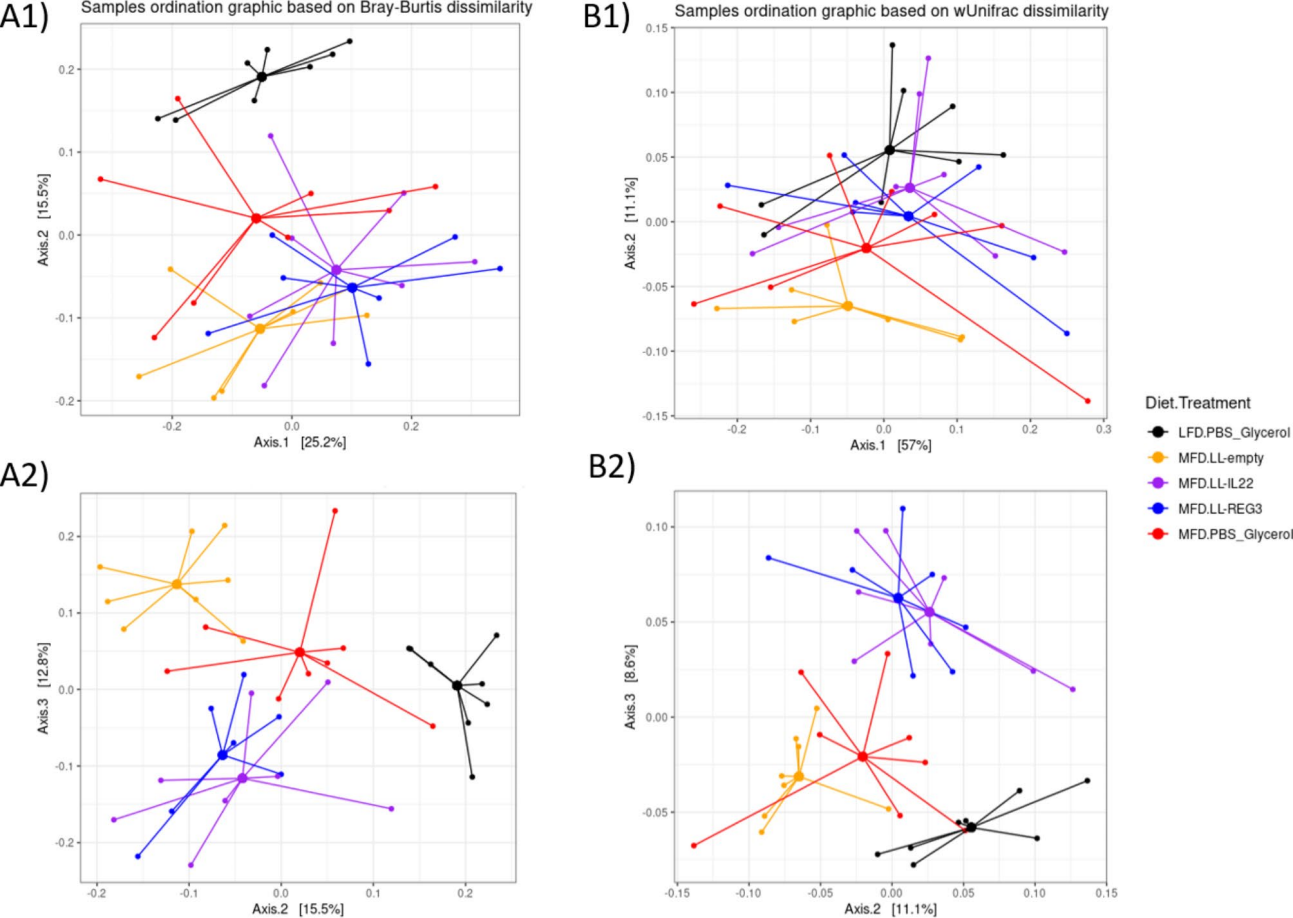
Principal coordinate analysis of microbial communities at OTU level. Based on (**A**) Bray-Curtis and (**B**) Weighted UniFrac dissimilarities.

Principal component analysis on the OTUs showed that diet and supplementation with LL-Probi-H1:empty, LL-Probi-H1:REG3A and LL-Probi-H1:IL22 were impactful on gut community composition (Fig. 5). Each group treatment clusterises separately from the others except the LL-Probi-H1:REG3A and LL-Probi-H1:IL22 groups which seem to clusterise together. PERMANOVA analysis on Bray-Curtis and weighted UniFrac distances show statistically significant differences at the OTU level depending on diet (Bray-Curtis : R2 = 0.11534; pval < 0.001 – wUniFrac : R2 = 0.06152, pval = 0.045), treatment (Bray-Curtis : R2 = 0.19302; pval < 0.001 – wUniFrac : R2 = 0.14069, pval = 0.035) and ALAT in blood plasma (Bray-Curtis : R2 = 0.05032; pval < 0.001 – wUniFrac : R2 = 0.06861, pval = 0.029; see Table 1).

**Table 1:** PERMANOVA analysis on Bray–Curtis and weighted UniFrac distances testing the correlation of diet, treatment and transaminase levels with OTUs composition in fecal microbiota.

	Df	SumsOfSqs	MeanSqs	F.Model	R2	Pr(> F)
PERMANOVA based on Bray-Curtis distance						
Diet	1	0.4636	0.46356	6.0175	0.11534	0.001 ***
Treatment	3	0.7758	0.25859	3.3567	0.19302	0.001 ***
Alat	1	0.2023	0.20225	2.6254	0.05032	0.001 ***
Asat	1	0.1124	0.11242	1.4593	0.02797	0.138
Residuals	32	2.4651	0.07703		0.61335	
Total	38	4.0191			1.00000	
PERMANOVA based on weighted UniFrac distance						
Diet	1	0.08553	0.085533	2.8503	0.06152	0.045 *
Treatment	3	0.19562	0.065205	2.1729	0.14069	0.035 *
Alat	1	0.09539	0.095388	3.1787	0.06861	0.029 *
Asat	1	0.05356	0.053556	1.7847	0.03852	0.120
Residuals	32	0.96028	0.030009		0.69066	
Total	38	1.39037			1.00000	

At the genus level we see a similar distribution of samples depending on group treatment (data not shown). PERMANOVA analysis on Bray-Curtis and weighted UniFrac distances shows a statistically different genus composition depending on diet (Bray-Curtis : R2 = 0.11067; pval < 0.001), treatment (Bray-Curtis : R2 = 0.20008; pval < 0.001 – wUniFrac : R2 = 0.13649, pval = 0.046) and ALAT in blood plasma (Bray-Curtis : R2 = 0.05623; pval < 0.009– wUniFrac : R2 = 0.07205, pval = 0.038; see Table 2).

**Table 2:** PERMANOVA analysis on Bray-Curtis and weighted UniFrac distances testing the correlation of diet, treatment and transaminase levels with genus composition in fecal microbiota.

	Df	SumsOfSqs	MeanSqs	F.Model	R2	Pr(> F)
PERMANOVA based on Bray-Curtis distance						
Diet	1	0.29396	0.293959	5.8376	0.11067	0.001 ***
Treatment	3	0.53145	0.177151	3.5180	0.20008	0.001 ***
Alat	1	0.14936	0.149355	2.9660	0.05623	0.009 **
Asat	1	0.07010	0.070104	1.3922	0.02639	0.199
Residuals	32	1.61139	0.050356		0.60664	
Total	38	2.65626			1.00000	
PERMANOVA based on weighted UniFrac distance						
Diet	1	0.07892	0.078923	2.7824	0.06034	0.052 .
Treatment	3	0.17853	0.059510	2.0980	0.13649	0.046 *
Alat	1	0.09425	0.094252	3.3228	0.07205	0.038 *
Asat	1	0.04867	0.048674	1.7160	0.03721	0.134
Residuals	32	0.90767	0.028365		0.69391	
Total	38	1.30805			1.00000	

**Fig. 6:**
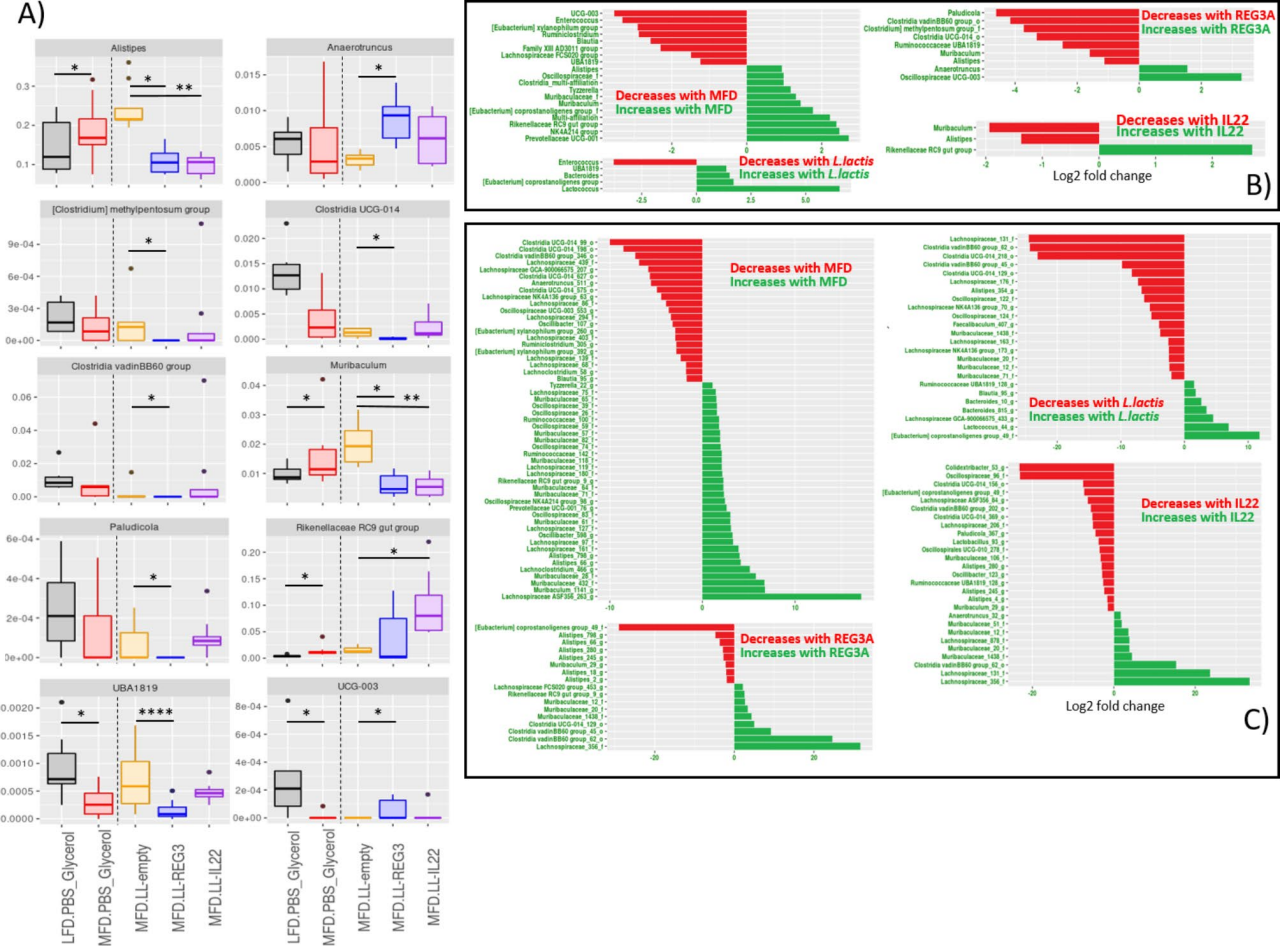
Significant differences in the microbiota composition at the genera or OTU level. (**A**) Relative abundance of the genera which are significantly increased or decreased between groups treated with *L.lactis* delivering plasmids for the expression of REG3A or IL22 and the empty *L. lactis* control group, (**B**) Differentially expressed genera between groups expressed in log 2 fold change and (**C**) Differentially expressed OTUs between groups expressed in log2 fold change.

### REG3A and IL-22 reverse*Alistipes* and *Muribaculum* increase due to MFD

From LFD to MFD control we see a strong relative increase of many genus among which some are significant such as *Muribaculaceae* (from 14.72 to 22.05%), *Rikenellaceae* (*Alistipes* went from 14.48 to 18.85% and *Rikenellaceae RC9 gut group* went from 0.42% to 1,44%), [Eubacterium] coprostanoligenes group, *Oscillospiraceae* NK4A214 group and *Tyzzerella*. Decreasing taxa include *Alloprevotella* (albeit non significantly but went from 12,78% to 4,36%, Fig. 6A, detailed in supplementary Table 1), Parabacteroides (from 4,93% to 1,92%), *Erysipelotrichaceae*, *Clostridia* UCG-014, *Anaerovoracaceae*, many *Lachnospiraceae* (*Lachnospiraceae FCS020 group*, *Ruminococcaceae UBA1819*, *Ruminiclostridium*, *Oscillospiraceae UCG-003*, *[Eubacterium] xylanophilum group*, and *Blautia*) and *Enterococcus* (Fig. 6B, detailed in supplementary Table 2). At the OTU level (Fig. 6.C, detailed in supplementary Table 3) 32 taxa are significantly increased (18 Firmicutes and 13 Bacteroidota) and 21 taxa are significantly decreased (all of which belong to the phylum Firmicutes). OTUs increased include 10 *Muribaculaceae* (including *Muribaculum*), 9 *Lachnospiraceae* (including *Tyzzerella*), 2 *Ruminococcaceae*, 7 *Oscillospiraceae*, 2 *Allistipes*, 1 *Rikenellaceae RC9 gut group* and 1 *Prevotellaceae UCG-001*. OTUs decreased include 4 *Clostridia UCG-014*, 12 *Lachnospiraceae*, 2 *Oscillospiraceae*, 1 *Clostridia vadinBB60 group*, 1 *Ruminococcaceae* and 1 *Ruminiclostridium*.

Effect of *L. lactis* administration is detectable in feces by sequencing. It increases *Ruminococcaceae UBA1819*, *Bacteroides* and *[Eubacterium] coprostanoligenes* group but decreases *Enterococcus* (Fig. 6B). At the OTU level 17 taxa are significantly decreased (12 Firmicutes and 5 Bacteroidetes) and 7 are increased (5 Firmicutes and 2 Bacteroidota). Among the decreased OTU we find 2 *Clostridia vadinBB60 group*, 2 Clostridia UCG-014, 5 *Lachnospiraceae*, 4 *Muribaculaceae*, 2 *Oscillospiraceae*, 1 *Faecalibaculum* and 1 *Alistipes* (Fig. 6). Most of these differentially expressed OTU have a low baseline of expression (< 100 mean reads). Hence, *Alistipes* is globally increased (but non-significantly) in this group compared to MFD control (24,42% against 18,85%, Supplementary Table 3) even if the only OTU to be differentially expressed by itself is decreased.

REG3A and IL22 have similar effects on gut microbiota composition with a significant decrease of *Alistipes* (from 24,42% to respectively 10.91 and 9,98%) and *Muribaculum* (from 2% to 0.62% and 0.57%) and an increase in Rikenellaceae RC9 gut group (from 1,55% to 4,02% and 10% respectively), *Oscillospiraceae* UCG-003 and *Anaerotrunctus* compared to LL-Probi-H1:empty. There was also an increase in total *Muribaculaceae* (from 14,73% in LL-probi-H1:empty to 20.67% in LL-Probi-H1:REG3A and 18,11% in LL-Probi-H1:IL22, see Fig. 6).

From LL-Probi-H1:empty to LL-Probi-H1:REG3A 9 OTUs are increased including 4 *Muribaculaceae*, 3 *Lachnospiraceae*, 1 *Clostridia vadinBB60* and 1 *Anaerotrunctus*. 18 OTUs are decreased including 3 *Alistipes*, 3 *Oscillospiraceae*, 2 *Clostridia* UCG-014, 2 *Muribaculaceae* (including *Muribaculum*), 2 *Ruminococcaceae*, 2 *Lachnospiraceae*, 1 *Clostridia vadinBB60 group*, 1 *[Eubacterium] coprostanoligenes group*, 1 *Oscillospirales UCG-010* and 1 *Lactobacillus* (Fig. 6).

From LL-Probi-H1:empty to LL-Probi-H1:IL22 9 OTUs are increased including 3 *Muribaculaceae*, 2 *Lachnospiraceae*, 2 *Clostridia vadinBB60 group*, 1 *Rikenellaceae* and 1 *Clostridia UCG-014*. 8 OTUs are decreased including 6 *Alistipes*, 1 *Muribaculum* and 1 *[Eubacterium] coprostanoligenes group* (Fig. 6).

## Discussion

The aim of this study, is to compare long time oral administration of IL-22 and REG3A in the context of HFD using recombinant lactic acid bacteria. We show that IL-22 and REG3A have opposite effects on fat accumulation in the liver and insulin resistance development despite inducing a similar shift in fecal microbiota composition.

Firstly, we find that while administration of LL-Probi-H1:empty in itself positively impacts weight gain and hepatic steatosis, this treatment was associated with lower food intake and did not prevent elevation of liver damage markers in the blood. Other studies, including ours, have shown similar results. In 2007, Bermudez-Humaran et al. demonstrated the effect of *L.lactis* NZ9000, a derivative of *L.lactis* MG1363, on weight gain in ob/ob mice^23^. This property was also described in obese mice after HFD^24,25^.

0Weight gain was significantly lower in LL-Probi-H1:REG3A treated mice compared to LL-Probi-H1:empty mice despite similar food intake, suggesting that REG3A’s effect is independent from the probiotic strain’s effect and food intake (Fig. 1). Interestingly, REG3A positive effects on hepatic steatosis are mitigated by elevated insulin resistance (Fig. 2). This effect is not significant compared to LL-Probi-H1:empty. Reg3 lectins are generally found to be downregulated in the intestine of obese animals^26^ and are protective against alcoholic steatohepatitis by reducing mucosa-associated microbiota and preventing bacterial translocation to the liver^27^. In 2018, S. Bluemel et al. found that Reg3β-deficient mice were protected against glucose intolerance but not liver disease in a Western-style fast food diet^28^. Our results seem to show a lack of protective effect from Reg3 lectins on oral glucose response in the prediabetic stage, contrasting with previous findings.

Modification of the composition of the gut microbiota is a key component of Reg3 lectins benefits on health as they play a major role in the maintenance of the separation between commensal bacteria and the epithelium^4^. In our study we found that REG3A impacted gut microbiota by strongly decreasing *Alistipes* and *Muribaculum* which were favored by MFD (Fig. 6). IL22 treatment, which is known to induce Reg3 lectins, had very similar effects on gut microbiota composition. Hendrixx et al. demonstrated that IL22 treatment improved NAFLD in wild-type mice, but not in Reg3y-/-, which linked the benefits of this cytokine to antimicrobial peptide production and gut microbiota^29^. Furthermore Reg3 lectins decrease associated with IL22 and IL33 deficiency was correlated with *Alistipes* increase in previous studies^15,30^ and IL22 deficient mice microbiota transfer was found to be colitogenic.

Paradoxically, in our study, IL22 treatment had opposite effects in respect of REG3A. Steatohepatitis was undoubtedly aggravated in LL-Probi-H1:IL22-treated mice as shown by histological cuts and elevated plasma ALAT (Fig. 3). Triglyceride levels in blood were paradoxically decreased. Such decrease has been previously observed in obese C57BL/6 mice and are likely due to either an increased triglyceride clearance or a suppression of triglyceride production by the liver^31^. Low-fasting triglycerides levels in the serum seems to be a marker of advanced liver disease in humans^32^. Three mice in the control MFD group and one mouse from the LL-Probi-H1:IL22 treated group had such extreme liver damage that cholesterol production was impaired (Fig. 3). However, in general, the LL-Probi-H1:IL22 had significantly lower insulin tolerance compared to LL-Probi-H1:empty-treated mice (Fig. 2). This seems coherent with multiple studies demonstrating the beneficial effects of IL22 on insulin resistance^12,14^. Our findings directly contradict previous studies in which administration of *Lactobacillus reuteri* engineered to produce IL22 alleviated steatohepatitis through upregulation of Reg3β and Reg3γ in mice intestines and was linked to microbiota regulation and lower bacterial translocation to the liver^19,29^. This discrepancy could be explained by the difference in treatment time. Indeed, in our experiment, we started treatment with LL-Probi-H1:IL22 administration at the same time as diet shift while Oh J-H et al. waited a month after the start of high-fat diet before treatment with recombinant *L. reuteri* began. Furthermore, our system induces endogenous expression of IL22 by host cells while their approach is exogenous which probably impacts dose-response and entry of the drug in the circulatory system.

IL22 promotes the proliferation and regeneration of tissues and has anti-apoptotic effects, which is why it is generally considered beneficial and is investigated as a promising drug for epithelial cell injury and alcoholic hepatitis^33^. However, in some cases, its sustained expression can lead to chronic inflammation, tissue damage and tumor formation, making it a double-edged sword^34,35^. Elevated IL22 expression in patients is correlated with incidence of type-2 diabetes mellitus^36^ and this cytokine takes up pathological functions in chronic liver inflammation depending on the recruitment of Th17 cells^37^. IL-17/IL-22-producing CD4 + T cells were found to infiltrate the liver and to be responsible for metabolically abnormal insulin-resistant obesity^38^. Moreover, IL22 amplifies IL1β expression in the adipose tissue which drives inflammation in NAFLD^10,39^.

We show thus that IL22 and REG3A have opposite effects on fat accumulation in the liver and insulin resistance development despite inducing a similar shift in fecal microbiota composition. Because the phenotypes expressed by the REG3A and IL22 groups are so different, despite a very similar effect of the administered bacteria on their microbiota composition, it is difficult to draw conclusions about the role of the microbiota in their harmful or protective effect. Although we did not test the microbiota of our treated mice by fecal transfer for confirmation, it is possible that any benefit brought on by IL22 through microbiota regulation was overridden by pro-inflammatory mechanisms due to long term overexpression of this cytokine. In the meantime, prudence is required while investigating the potential use of IL22 as a therapeutic drug.

## Methods

### Mice breeding conditions and food

C57BL/6JRj mice were purchased from Janvier Labs and kept in compliance with the institutional and European Union guidelines for laboratory animal care. The study was approved by the COMETHEA ethics committee (n°2015070115416973). This study is reported in accordance with ARRIVE guidelines. They were delivered at 6 weeks of age and co-housed by groups of four mice per cage in an air-conditioned room with controlled temperature and circadian cycle (12 h light, 12 h dark) in the Animal Facility of the National Institute of Agronomic and Environment Research.

After one week of acclimatization, mice were assigned to groups and fed either a low fat control diet (10.4% energy from fat; TD.120508, Purified Control (93 M, Teklad VM), Envigo) or a milkfat diet (37.4% energy from fat; TD.97222, 18% Milkfat Diet, Envigo) *ad libitum* for 12 weeks during which mice weight and food intake were measured once a week. Experimental diets were treated with UV and stored at 4 °C in airtight containers until use.

Mice were divided into five different groups (*n* = 16 for each): low fat diet control (LFD), milkfat diet control (MFD), MFD + 10^9^ CFU LL-Probi-H1:empty, MFD + 10^9^ CFU LL-ProBi-H1:REG3A or MFD + 10^9^ CFU LL-Probi-H1:IL22. Bacteria were administered daily by intragastric gavage for the duration of the experiment (see Fig. 1A).

### IL-22 and REG3A expressing plasmids

LL-Probi-H1:empty carries the Probi-H1 plasmid which allows for the expression of proteins of interest in eukaryotic cells under the control of pCMV as described by Meynier et al.^20^. REG3A and IL-22 sequences were cloned into the plasmid expression cassette which resulted in the Probi-H1:REG3A and Probi-H1:IL-22 plasmids. Plasmids were then inserted into *L. lactis* MG1363 which was used as the delivery vector for DNA transfer.

### Oral glucose tolerance test

Mice were fasted for 16 h. Glucose was administered orally at 2 g/kg and glycemia was measured at 0, 15, 30, 60 and 120 min after administration. For measurements, Accu-Chek Performa Nano blood glucose monitoring strips (Cat #06454011031) and Accu-Check Performa blood glucose meter (Roche Diabetes Care) were used. Blood was collected with EDTA-coated capillary tubes (Microvette^®^ CB 300 K2E, Sarstedt), centrifuged for 10 min at 1000 g to isolate plasma and glucose-stimulated insulin concentration (GSIC) over time 0 to 30 min was measured with Rat/Mouse Insulin ELISA kit (EZRMI-13 K, Sigma-Aldrich). For stimulation, 200µL of a solution of 30% glucose was administered orally to mice.

### Insulin tolerance test

Mice were fasted for 4 h. Insulin was injected intraperitoneally (0.5 mU/g body weight) and glycemia was measured at 0, 15, 30, 60 and 120 min after administration. For measurements, Accu-Chek Performa Nano blood glucose monitoring strips (Cat #06454011031) and Accu-Check Performa blood glucose meter (Roche Diabetes Care) were used.

### Necropsy, tissue and blood sampling

Mice were euthanized by cervical dislocation. Blood was sampled from the mandibular vein before necropsy in Gel + Lithium Heparin tubes (Cat #812550, Kima) and subsequently centrifuged at for 10 min at 1000 g to isolate plasma from red blood cells and stored at -80 °C. Concentrations of triglycerides (TG), total cholesterol, high-density lipoprotein (HDL), aspartate aminotransferase (ASAT) and alanine aminotransferase (ALAT) were determined at the Laboratoire de biochimie of Institut Claude Bernard with the Olympus AU400 Chemistry Analyzer. Tissues were removed and one piece was kept in RNA latter at room temperature for half a day then stored at -80 °C, one piece was put in paraformaldehyde for histologic analysis and one piece was immediately snap frozen in liquid nitrogen and stored at -80 °C until further processing.

### Liver histology and hepatic lipids measurement

A slice of the left lobe of the liver was sectioned and fixed in 4% PFA for 48 h and transferred into 70% ethanol. It was then fixed in paraffin, trimmed, processed and cut into slices which were approximately 3 μm thick, mounted on a glass slide and stained with H&E. Hepatic lipids were quantified blindly with the ImageJ software as described before^40^.

### DNA extraction and 16 S DNA sequencing

DNA was extracted using the protocol of Godon et al.^41^. DNA concentration and purity were determined using a NanoDrop instrument. We used the MTP™ Taq DNA Polymerase Kit (Sigma Cat#D7442-250UN) with 10 ng of extracted DNA for PCR amplification of the V3-V4 region of 16 S rDNA. The amplification was performed with the primers MSQ-16SV3F (CTTTCCCTACACGACGCTCTTCCGATCTACGGRAGGCWGCAG) and MSQ-16SV4R (GGAGTTCAGACGTGTGCTCTTCCGATCTTACCAGGGTATCTAATCCT). PCR conditions were: 94 °C for 1 min; 30 cycles at 94 °C for 1 min, 65 °C for 1 min, and 72 °C for 1 min; and a final extension of 72 °C for 10 min. Amplification products were checked via electrophoresis on 2% agarose gel. Sequencing was performed using the Illumina Miseq (250 × 2 bp) system.

### Analysis of sequencing data

16 S sequencing data were uploaded to the Galaxy platform^42,43^ at https://galaxy.migale.inra.fr/. We used the FROGS pipeline to produce abundance tables of operational taxonomic units (OTUs). Denoising and clustering were performed with SWARM, chimera removal with VSEARCH, and affiliation with RDP Classifier using the 16S_SILVA_Pintail100_138 database. OTUs with a minimum presence of 0.005% in all sequences were kept. OTUs were filtered for a minimum identity of 97% and a minimum coverage of 95%. Statistical analysis of results was performed using the phyloseq package on RStudio version 4.0.3. Measures of α- (Observed, Chao1, ACE, Shannon and Inverse Simpson indexes) and β-diversity (Bray-Curtis and weighted UniFrac dissimilarity score) were calculated. Relative abundance table was written on rarefied data at the genus level. Analysis of differential expression was performed on raw data with the DESeq2 package at the genus and OTU levels.

### Statistical analysis

For data with a normal distribution, one-way ANOVA or two-way ANOVA was performed. Otherwise, Mann-Whitney analysis was performed. *p* values < 0.05 were considered statistically significant.

## Electronic supplementary material

Below is the link to the electronic supplementary material.

Supplementary Material 1

Supplementary Material 2

Supplementary Material 3

Supplementary Material 4

## Data Availability

The datasets generated and/or analysed during the current study are available in the NCBI repository, https://www.ncbi.nlm.nih.gov/sra/PRJNA1170054.
