# Supplementary material for: REG3A and IL22 have opposite effects on fat accumulation in the liver in a high-fat diet
Source: Sci Rep. 2025 Sep 30;15:33733. doi: 10.1038/s41598-024-84700-w (PMC12485020; doi:10.1038/s41598-024-84700-w)
Supplement: Supplementary file 4 — Supplementary Material 4 [file 41598_2024_84700_MOESM4_ESM.pptx]

## Slide 1
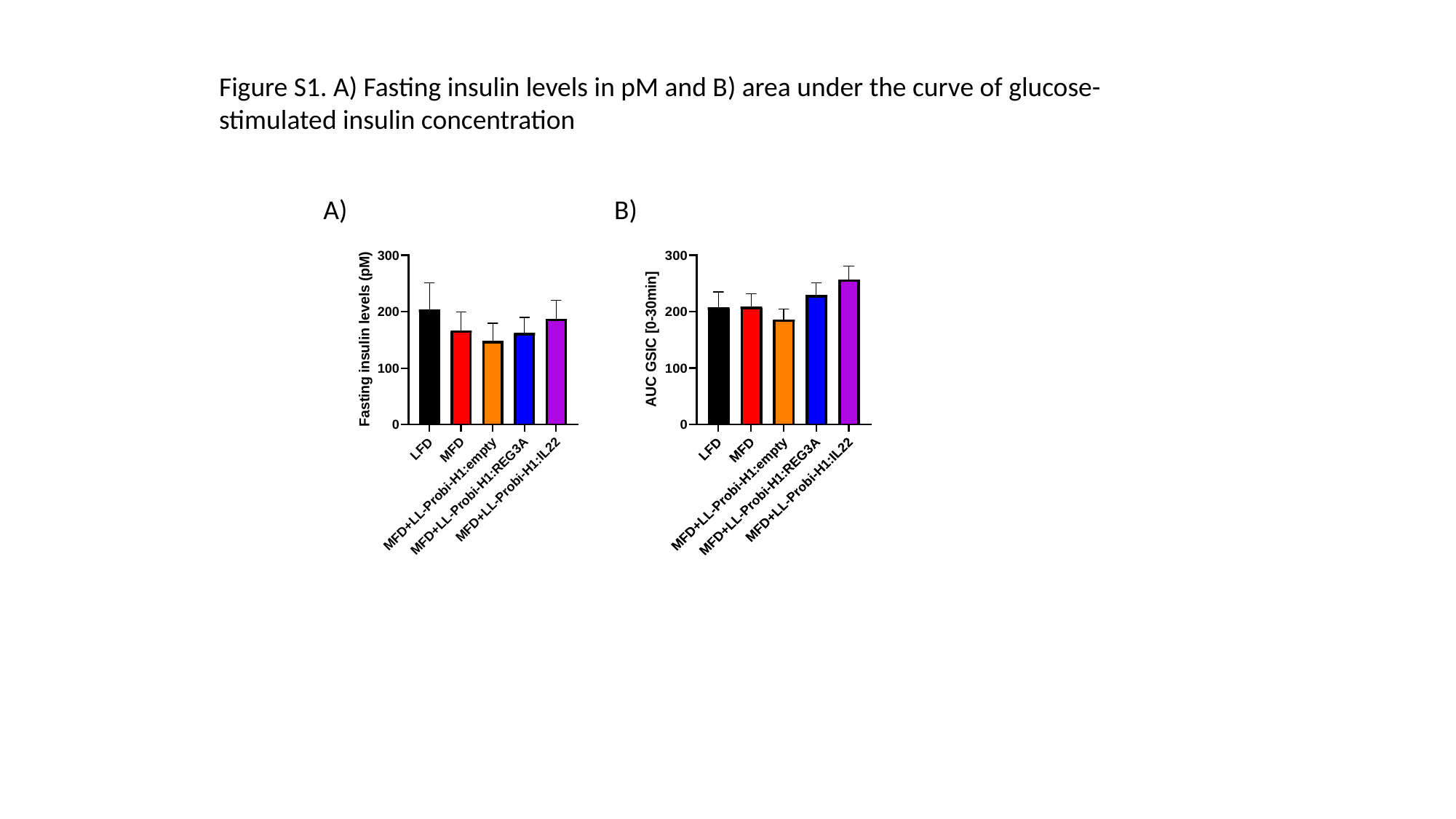

Figure S1. A) Fasting insulin levels in pM and B) area under the curve of glucose-stimulated insulin concentration
A)
B)
